# Functional Relationships of Wood Anatomical Traits in Norway Spruce

**DOI:** 10.3389/fpls.2020.00683

**Published:** 2020-05-26

**Authors:** Alma Piermattei, Georg von Arx, Camilla Avanzi, Patrick Fonti, Holger Gärtner, Andrea Piotti, Carlo Urbinati, Giovanni Giuseppe Vendramin, Ulf Büntgen, Alan Crivellaro

**Affiliations:** ^1^Department of Geography, Faculty of Earth Sciences and Geography, University of Cambridge, Cambridge, United Kingdom; ^2^Swiss Federal Research Institute for Forest, Snow and Landscape Research (WSL), Birmensdorf, Switzerland; ^3^Department of Chemistry, Life Science and Sustainability, University of Parma, Parma, Italy; ^4^Institute of Biosciences and Bioresources, Italian National Research Council, Florence, Italy; ^5^Department of Agricultural, Food and Environmental Sciences, Marche Polytechnic University, Ancona, Italy; ^6^Global Change Research Institute, Czech Academy of Sciences, Brno, Czechia; ^7^Department of Geography, Faculty of Science, Masaryk University, Brno, Czechia

**Keywords:** allometric effect, ontogenesis, quantitative wood anatomy, temporal stability, xylem hydraulic constraints

## Abstract

The quantitative assessment of wood anatomical traits offers important insights into those factors that shape tree growth. While it is known that conduit diameter, cell wall thickness, and wood density vary substantially between and within species, the interconnection between wood anatomical traits, tree-ring width, tree height and age, as well as environment effects on wood anatomy remain unclear. Here, we measure and derived 65 wood anatomical traits in cross-sections of the five outermost tree rings (2008–2012) of 30 Norway spruce [*Picea abies* (L.) H. Karst.] trees growing along an altitudinal gradient (1,400–1,750 m a.s.l.) in the northern Apennines (Italy). We assess the relationship among each anatomical trait and between anatomical trait groups according to their function for (i) tree-ring growth, (ii) cell growth, (iii) hydraulic traits, and (iv) mechanical traits. The results show that tree height significantly affects wood hydraulic traits, as well as number and tangential diameter of tracheids, and ultimately the total ring width. Moreover, the amount of earlywood and latewood percentage influence wood hydraulic safety and efficiency, as well as mechanical traits. Mechanically relevant wood anatomical traits are mainly influenced by tree age, not necessarily correlated with tree height. An additional level of complexity is also indicated by some anatomical traits, such as latewood lumen diameter and the cell wall reinforcement index, showing large inter-annual variation as a proxy of phenotypic plasticity. This study unravels the complex interconnection of tree-ring tracheid structure and identifies anatomical traits showing a large inter-individual variation and a strong interannual coherency. Knowing and quantifying anatomical variation in cells of plant stem is crucial in ecological and biological studies for an appropriate interpretation of abiotic drivers of wood formation often related to tree height and/or tree age.

## Introduction

**FIGURE 1:**
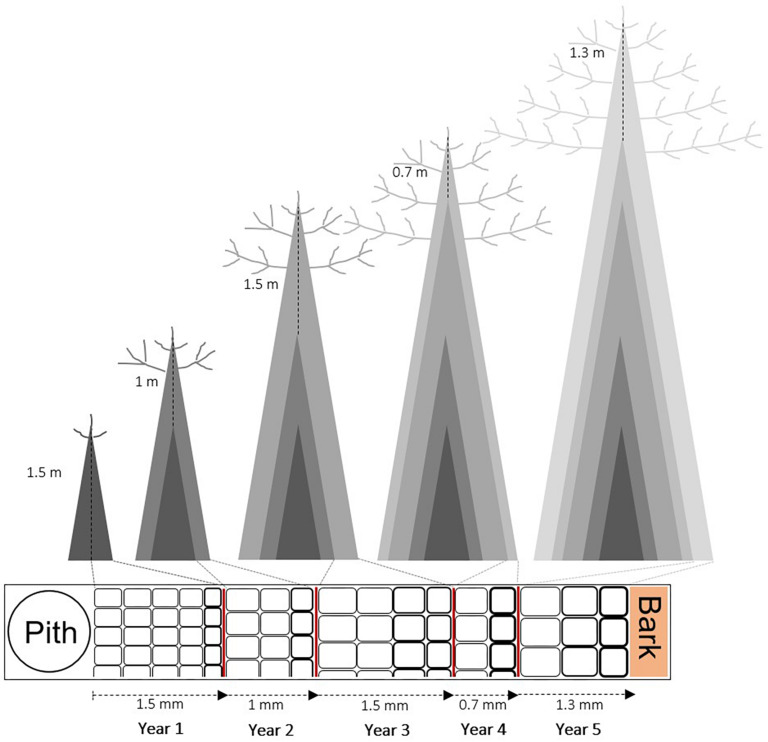
Sketch of the wood structure scaling from pith-to-bark in relation to tree height (modified from Rosell et al., 2017) in a hypothetical 5-years-old tree, supposing the same radial (tree-ring width) and longitudinal (internode length) growth. The change in number and size of tracheids is shown from pith to bark. There are more and smaller tracheids in short trees (or close to the pith), and fewer and wider tracheids in tall trees (or close to the bark) compared to the previously formed ring.

Every single cell in a tree ring is derived from the division of a cambial cell (periclinal growth), which then expands to its final size, thickens its cell wall and eventually lignifies before programmed cell death (Plomion et al., 2001). These steps are triggered and guided by the complex interplay of genetic factors, ontogenesis (organism development over time), hormone regulation, and environmental conditions (Aloni, 2013). Wood cell formation, therefore, represents a unique and complex combination of interacting processes resulting in a complete tree ring (Vaganov et al., 2006; Fonti and Jansen, 2012). An extensive literature faced the task of relating the wood cellular structure to its functioning (e.g., Tyree and Ewers, 1991; Domec et al., 2009; Fonti et al., 2013; Hacke et al., 2015; Beeckman, 2016; De Micco et al., 2019). For example, tracheids diameter and density, earlywood–latewood percentage, and cell wall thickness (CWT) have been demonstrated to play a central role in regulating tree hydraulic efficiency and mechanical functioning (e.g., McCulloh et al., 2010; Rosner, 2013; Prendin et al., 2018), but also to be strongly influenced by climate (e.g., Larson, 1994; Carrer et al., 2016; Castagneri et al., 2017). Differently, some hydraulic parameters, such as hydraulic diameter and specific hydraulic conductivity, are influenced by tree height (e.g., Anfodillo et al., 2013; Rosell et al., 2017). However, inter- and intra-individual variation of multiple wood anatomical traits have been rarely analyzed on large numbers of trees growing under different environmental conditions (Rungwattana and Hietz, 2017; Prendin et al., 2018). As a consequence, it is still unclear how relevant is the contribution of each factor balancing the trade-off at the wood cell, and consequently, at the tree-ring level. Dendrophenotypic traits (e.g., Evans et al., 2018; Heer et al., 2018) and the development of optimal strategies for quantifying their variation are important for assessing the effect of potential drivers of individual variation in growth traits. Recently, several studies brought a new perspective on the inter-individual variation of wood anatomical traits highlighting the role of tree height as the driver of wood anatomical variation along plant stem, as opposed to the classical pith-to-bark age trend (Enquist, 2002; Olson et al., 2014; Kašpar et al., 2019). This is especially true for conduits in woody species, designed to transport water from roots to leaves. According to fluid dynamics, the increase in plant height would be accompanied with a concomitant increase in hydrodynamic resistance along the conduit (West et al., 1997). This effect could constrain height growth of a plant, unless it is compensated by a dimensional increase of wood conduits during ontogenesis. It has been repeatedly observed that conduit diameter becomes larger (and longer) toward the plant base as the distance to the tree-top increases, a pattern called “conduit widening” (Anfodillo et al., 2013; Lazzarin et al., 2016; Rosell et al., 2017). The conduit widening, when viewed on a wood cross-section from the stem base, is represented by an increase in conduit size from pith-to-bark (as described by Malpighi, 1675) and it is often interpreted as an age-related trend (Gartner, 1995; Lachenbruch et al., 2011). In a short plant bearing narrow conduits, the increase in conduit size from pith-to-bark is a consequence of the conduits getting larger to compensate for a height increase (Carrer et al., 2015) (Figure 1).

To investigate the synergy of tree height, age, and environmental growing conditions on wood anatomical traits we quantified wood anatomical variation in cross-sections of Norway spruce, a conifer species featuring a simple wood structure where tracheids have both conductive and mechanical functions (Evert, 2006). The aims of this paper are to investigate (1) how tracheid anatomy in radial and tangential directions is related to ontogenesis, (2) how wood anatomical variation reflects functional constraints and trade-offs, and (3) how wood anatomical traits vary at the inter-individual and inter-annual level. To minimize the climate influence on wood anatomical traits, considered “noise” in this study, only the five outermost tree rings (2008–2012) were analyzed. The results provided a summary of wood anatomical trait variability and suggested possible implications for related research fields such as plant ecology and evolutionary genetics.

## Materials and Methods

### Study Site and Samples Collection

Field sampling was carried out in 2012 in the Campolino Natural Reserve (northern Apennines) (Table 1), which is the southernmost known autochthonous population of Norway spruce [*Picea abies* (L.) H. Karst.] in Italy (Chiarugi, 1936; Magri et al., 2015; Avanzi et al., 2019). Three plots were established along an altitudinal transect on the same slope. The high altitude plot (CAM-H, ∼1,730 m a.s.l.) is a pure, dense and relatively young spruce forest recolonizing an old pasture. The intermediate plot (CAM-E, ∼1,615 m a.s.l.) is a mature mixed forest with Norway spruce, silver fir (*Abies alba* Mill.) and European beech (*Fagus sylvatica* L.). The lowest plot (CAM-L, ∼1,475 m a.s.l.) is a similar mixed forest, but with less spruce (Table 1). In each plot sampled, trees were labeled and georeferenced with a geographic positioning system device (GPS; Garmin, 2 m resolution). Stem diameter at breast height (DBH) and tree height were measured, and one increment core was collected with a 5 mm Pressler borer at DBH in cross-slope direction to avoid compression wood.

**TABLE 1:** Geographic coordinates and stand characteristics of the three Norway spruce [*Picea abies* (L.) H. Karst.] plots.

Code	Latitude (°N)	Longitude (°E)	Elevation (m a.s.l.)	Area (ha)	Density (trees ha^–1^)	Age (years) (min–max) ± SD	Tree height (m) (min–max) ± SD	DBH (cm) (min–max) ± SD
CAM-H	44°06′36″	10°39′44″	1,730	0.25	648	47 (34–71) ± 11	14 (10.5–18) ± 3	34 (22–56) ± 10
CAM-E	44°06′47″	10°39′47″	1,615	0.83	127	119 (35–151) ± 41	20 (11–27) ± 5	43 (20–67) ± 14
CAM-L	44°07′07″	10°40′18″	1,475	1.95	28	78 (39–155) ± 40	22 (13–33) ± 6	53 (28–122) ± 30

*Density refers only to Norway spruce trees in the sampling area, whereas the mean, min, max, and standard deviation (SD) of age, tree height and DBH refer to the 30 trees analyzed in this study. (For the measurements of Age and DBH of the three plots see Avanzi et al., 2019).*

The three plots fall in the same spatial grid, so monthly mean temperatures and total precipitation for the 2008–2012 period were obtained from the CRU TS V.4.0 (land) 0.5° grid (KNMI Climate Explorer^1^).

### Tree Selection and Wood Anatomical Traits

A total of 10 trees per plot (Table 1, Supplementary Figure S1) were selected to maximize variation in tree height, age and DBH (Supplementary Figure S2). Wood microsections were prepared to collect high-resolution anatomical images following the standard protocols (Gärtner and Schweingruber, 2013; von Arx et al., 2016; Prendin et al., 2017). From each core, 12–15 μm thick cross-section from the last five tree rings (dated 2008–2012) were cut using a WSL lab-microtome (Gärtner et al., 2015a, b). All sections were double-stained using Safranin and Astra blue, dehydrated with ethanol, and permanently fixed on a microscope slide with Canada balsam. Overlapping images of cross-sections were captured at 100 times magnification using a digital camera (Canon EOS 650D, Canon Inc., Tokyo, Japan) through an Olympus BX41 microscope (Olympus Corp., Tokyo, Japan). PTGUI v10 (New House Internet Services B.V., Rotterdam, NL, United States) was used to stitch single images and produce a high-resolution composite image (2.36 pixels/μm). These images were then processed with the software ROXAS (v3.0.88, von Arx and Carrer, 2014), which provides a large set of measurements of tree-ring width (TRW) and wood anatomical traits (Table 2). Image analysis and anatomical data collection was restricted to a width of one tangential millimeter within each tree ring.

**TABLE 2:** List of all wood anatomical traits used in this study, dividing by their function.

Functional traits	Parameters	Acronym	Unit	Measured/derived	Portion within ring	Explanation of parameters	References
Ring growth	Tree-ring width	TRW	μm	M	All ring		
	Basal area increment	BAI	mm^2^year^–1^	M	All ring	*BAI*_*i*_ = ∏(∑*a*_*i*_)^2^−(∑*a*_*i*−1_)^2^	
	Percentage of EW	EW%	%	D	All ring		
	Percentage of LW	LW%	%	D	All ring		
	Ratio between LW and EW	LW%/EW%	/	D	All ring		
Cell growth	No. cells per mm in tangential direction	NoCells_tang	No.	M	LW		
	No. cells radial in direction	NoCells_rad	No.	D	All ring		
	Earlywood number of cells	EW_Nocells	No.	D	EW		
	Latewood number of cells	LW_Nocells	No.	D	LW		
	Earlywood width	EWW	μm	D	EW		
	Latewood width	LWW	μm	D	LW		
	Radial cell (lumen) diameter	Drad	μm	M	All ring		
	Tangential cell (lumen) diameter	Dtan	μm	M	All ring		
		Drad_EW	μm	D	EW		
		Dtan_LW	μm	D	LW		
	Cell wall area	CWA	μm^2^	M	All ring	Mean cell wall area per ring	
		CWA_EW	μm^2^	D	EW		
		CWA_LW	μm^2^	D	LW		
	Lumen area	LA	μm^2^	M	All ring		
		LA_01,25,50,75,99 percentile	μm^2^	D	Percentile	LA_01 = LW; LA_99 = EW	
	Cell density	CD	No./mm^2^	M	All ring	Global mean cell density	
	Number of total cells within the ring	CNO	No.	M	All ring	Number of cells	
		CNO_EW	No.	D	EW		
		CNO_LW	No.	D	LW		
Mechanical support	Cell wall thickness (radial and tangential)	CWT all	μm	D	All ring	Mean thickness of all cell walls [(CWTrad + CWTtan)/2]	Almeras and Gril (2007). *Tree Physiology* 27:1505–1516
		CWT all_EW	μm	D	EW		
		CWT all_LW	μm	D	LW		
		CWTall_01,25,50,75,99 percentile		D	Percentile	CWTall_99 = LW; CWTall_01 = EW	
	Cell wall thickness – tangential direction	CWT tan	μm	M	All ring	Mean thickness of tangential cell walls [(CWTpi + CWTba)/2]	
		CWT tan_EW	μm	D	EW		
		CWT tan_LW	μm	D	LW		
		CWTtan_01,25,50,75,99 percentile		D	Percentile	CWTtan_99 = LW; CWTtan_01 = EW	
	Cell wall thickness – radial direction	CWT rad	μm	M	All ring	Mean thickness of radial cell walls [(CWTle + CWTri)/2]	
		CWT rad_EW	μm	D	EW		
		CWT rad_LW	μm	D	LW		
		CWTrad_01,25,50,75,99 percentile		D	Percentile		
	Mork’s index	rTSR		D	All ring	Mean radial thickness to span ratio, Mork’s index per ring: ratio between 4× single cell wall thickness and tracheid diameter in radial direction	Denne (1988). *IAWA Bulletin* 10(1): 59–62
		rTSR_EW		D	EW		
		rTSR_LW		D	LW		
		rTSR_01,25,50,75,99 percentile		D	Percentile		
	Anatomical density	RWD		D	All ring	Mean relative anatomical cell density [CWA/(CWA + CA)] per ring	
		RWD_EW		D	EW		
		RWD_LW		D	LW		
		RWD_01,25,50,75,99 percentile		D	Percentile		
Hydraulic conductivity	Mean hydraulic diameter	Dh	μm	D	All ring	Mean hydraulic diameter per ring: [sum(2 × (cell lumen area/PI)^∧^0.5)^∧^5]/[sum(2 × (cell lumen area/PI)^∧^0.5)^∧^4]	Kolb and Sperry (1999). *Ecology* 80:2373–2384
	Specific hydraulic conductivity	Ks	m^2^ MPa^–1^ s^–1^	D	All ring	Xylem-specific potential hydraulic conductivity [m^2^ × s^–1^ × MPa^–1^] assuming a tube length of 1 m: Kh/Xylem area. (Xylem area in m^2^)	Tyree and Zimmermann (2002). Springer
	Theoretical hydraulic conductivity	Kh	m^3^ MPa^–1^ s^–1^	D	All ring	Accumulated potential hydraulic conductance [m^3^ × s^–1^ × MPa^–1^] as approximated by Poiseuille’s law and adjusted to elliptical tubes	Tyree and Zimmermann (2002). Springer
Hydraulic safety	Implosion safety	[t/b]^2^		D	All ring	Cell wall Reinforcement Index [t/b]^2^ per ring. “t” is the double cell wall thickness and b the length of the same cell wall; the smaller of the radial or tangential values is selected. (Low value of [t/b]^2^) means that a tree is more vulnerable to cavitation)	Hacke et al. (2001). *Oecologia* 126:457–461
		[t/b]^2^_05		D	Percentile	[t/b]^2^_05 = EW	
	Hydraulic carbon use efficiency	HCUE	kg m^–1^ MPa^–1^ s^–1^ μm^–2^	D	All ring	Hydraulic return for a given carbon investment	Prendin et al. (2018). *Functional Ecology* 1–15

*Some of them are an output of ROXAS, some are direct measurements and others are indexes. Mean hydraulic diameter (Dh – mean hydraulic diameter per ring), theoretical specific hydraulic conductivity (Ks – water transported per unit time per pressure gradient, normalized by its cross-sectional area), and theoretical hydraulic conductivity (Kh – water transported per unit time per pressure gradient) were used as proxies for hydraulic efficiency (McCulloh et al., 2010; Jyske and Hölttä, 2015). Mean tracheid cell wall reinforcement index ([t/b]^2^) was used as a proxy for hydraulic safety against cell implosion (Hacke et al., 2001), while “hydraulic carbon use efficiency” index (HCUE) was used as a proxy for the hydraulic return for a given carbon investment (Prendin et al., 2018). Additional anatomical metrics measured were total number of tracheids (CNO), number of tracheids in tangential (NoCells_tan), and radial direction (NoCells_rad), their lumen tangential and radial diameter (Dtan and Drad), their cell wall area (CWA) and lumen area (LA). Tree-ring width (TRW), EW and LW width (EWW and LWW) and percentage (EW% and LW%), as well as their ratio (LW/EW) were measured. The mechanical support function and carbon allocation of the xylem were estimated using mean cell wall thickness (CWT) in EW and LW, as well as relative wood density (RWD), and mean radial thickness to span ratio (Mork’s index) (RTSR). LA, CWT, RTSR, RWD, and [t/b]^2^ at different percentiles were also measured.*

The wood anatomical traits used in this study were direct measurements and derived from ROXAS output (Table 2). The analysis was performed within a tree ring, and in the earlywood (EW) and latewood (LW). TRW, EW and LW width (EWW and LWW), and their percentage (EW% and LW%) calculated as the ratio of EWW (LWW) to TRW, as well as their ratio (LW/EW) were measured. To tell apart EW and LW tracheids, ROXAS automatically computes the Mork’s index which is the ratio between twice the double CWT and the lumen diameter, both measured in radial direction (Denne, 1988) (Table 2). Additionally, the ratio between radial and tangential diameter of tracheid lumen was applied to discriminate between EW (ratio > 1) and LW (ratio < 1) tracheid shape. The number of tracheids in tangential direction was manually counted in the LW, and tracheid number in radial direction was computed by dividing the total number of tracheids in each tree ring (ROXAS output) by the number of tracheids in tangential direction within 1 mm strip. Wood anatomical traits were grouped in traits related to (i) tree-ring growth, (ii) cell growth, (iii) hydraulic efficiency and hydraulic safety, and (iv) mechanical support and carbon investment (Table 2).

### Statistical Analysis

#### Univariate and Multivariate Analysis of Wood Anatomical Traits

In order to assess the variability of wood anatomical traits, their attributed functions and the ontogenetic effect, univariate and multivariate analyses were performed. For assessing the pairwise relationships among wood anatomical traits, and the effect of tree height and tree age, linear, exponential and logarithmic functions were fitted, and the function with the highest *R*^2^ was chosen. The *R*^2^ of the logarithmic and exponential regressions were calculated by log-transforming the *x*- and *y*-axes, respectively. A principal component analysis (PCA) was carried out on each single year dataset using the *prcomp* function in R (R Core Team, 2018) to simultaneously describe the relationships among the 65 anatomical traits scored.

#### Identifying the Main Sources of Wood Anatomical Variation

The effects of tree height, age and plot (the latter used as a proxy for environmental variation along the altitudinal transect) on principal component (PC) scores summarizing wood anatomical variation, were assessed using a multiple regression approach through the *lm* function in R. The first three PC scores calculated on the 2012 dataset (when tree height data was available) were used as dependent variables in the multiple regression. For each PC, the *Anova* function in the R package *car* (Fox and Weisberg, 2011) was used to perform a backward model selection. A variable was dropped when the Likelihood Ratio Test on nested models indicated a simpler model structure as more parsimonious. Additionally, Mantel tests were performed between the matrix of pairwise geographic distances and the matrix of individual differences in PC scores to test whether spatial proximity determines more similar wood anatomical traits. Such analysis was performed at the plot level using the R package *vegan*. The effect of climatic variation was minimized by confining all analyses to the same five calendar years (2008–2012).

#### Assessing Inter-Annual Stability of Wood Anatomical Variation

A PCA was performed on each tree-ring dataset of wood anatomical traits relative to each year of the selected time period. To quantify the correlation of individual wood anatomical traits among years, a Procrustes test was performed on PC scores of different years using the *protest* function of the R package *vegan* (Oksanen et al., 2018). This statistical test measures the correlation between two point configurations (i.e., PC scores). Pairwise correlations between all possible combinations of years were visualized using the *procrustes* function, showing the amplitude and the direction of PC scores changes. Additionally, a coefficient of variation (CV) among years was calculated for each wood anatomical trait at the individual level. Individual CVs were then averaged among individuals to rank wood anatomical traits in terms of their stability through time.

## Results

### Inter-Individual Variation of Wood Anatomical Traits and Their Relationships: Univariate Analysis

**FIGURE 2:**
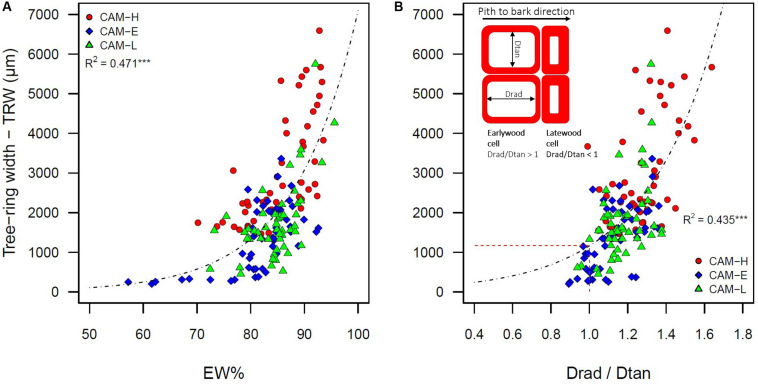
**(A)** Tree-ring width (TRW) of the five outermost tree rings as a function of earlywood percentage (EW%). **(B)** TRW as a function of the ratio between radial diameter (Drad) and tangential diameter (Dtan) of the tracheid lumen. Values of Drad/Dtan ratio >1 indicate that the shape of the tracheid is more EW-like. The inset figure represents a graphical sketch of earlywood and latewood tracheids as seen in a cross-section, describing the radial and tangential diameter, and their ratio. The dashed line represents the Drad/Dtan ratio equal to one. The three symbols (circle, diamond, and triangle) correspond to trees from CAM-H (red), CAM-E (blue), and CAM-L (green) plots. Both relationships were best described by an exponential function (***P < 0.001).

Tree-ring width was positively correlated with the number of tracheids per radial row (*r* = 0.99). TRW is EW width dependent (*R*^2^ = 0.993, *P* < 0.001), showing on average an EW% higher than 80% (Figure 2A). TRW was also positively related to the ratio between radial and tangential diameter (Drad/Dtan) of tracheid lumen (*R*^2^ = 0.435, *P* < 0.001) (Figure 2B). The lumen size maintained a proportional increase in radial and tangential diameter only in EW but not in LW tracheids (Supplementary Figure S3).

**FIGURE 3:**
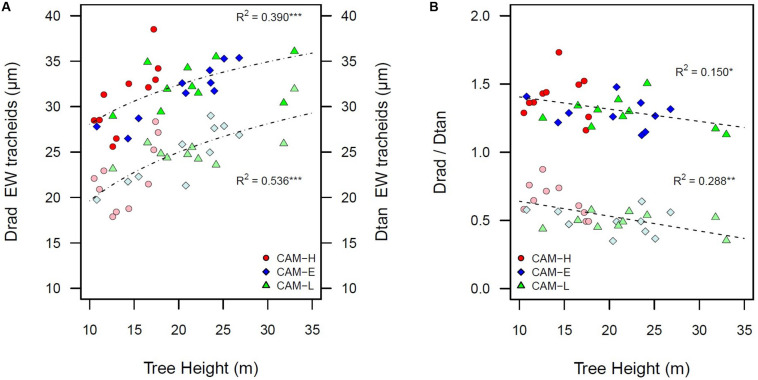
**(A)** Radial diameter (Drad) of EW tracheid lumen in bright color, and tangential diameter (Dtan) of EW tracheid lumen in pale color as a function of tree height. **(B)** Negative relationship between the ratio of radial and tangential diameter of tracheid lumen (Drad/Dtan) and tree height in bright color for EW portion and in pale color for the LW portion. The data shown are for the last year (2012). Each symbol (circle, diamond, and triangle) corresponds to a tree from the three plots CAM-H (red), CAM-E (blue), and CAM-L (green). All relationships are significant (*P < 0.05, **P < 0.01, ***P < 0.001).

Because tree height measurements prior to the year of sampling were not available, only Drad and Dtan of the outermost ring (2012) can be related to tree height. Dtan was more related to tree height than Drad, and these relationships were strongest for EW (EW Dtan: *R*^2^ = 0.536, EW Drad: *R*^2^ = 0.390, and LW Dtan: *R*^2^ = 0.357, *P* < 0.001) (Figure 3A). The Drad/Dtan scaled with tree height: taller trees had proportionally larger Dtan in EW (*R*^2^ = 0.530, *P* < 0.001), meaning that the tangential diameter increased more with tree height than Drad (Figure 3B). As a consequence, the tangential number of tracheids was negatively related to tree height (*R*^2^ = 0.473, *P* < 0.001).

Tracheid diameter affected both cell density (CD), the number of tracheids per mm^2^, and their lumen area (LA). Both CD and LA were related to tree height but with opposite directions: CD decreases with tree height, while LA increases (Supplementary Figure S4).

**FIGURE 4:**
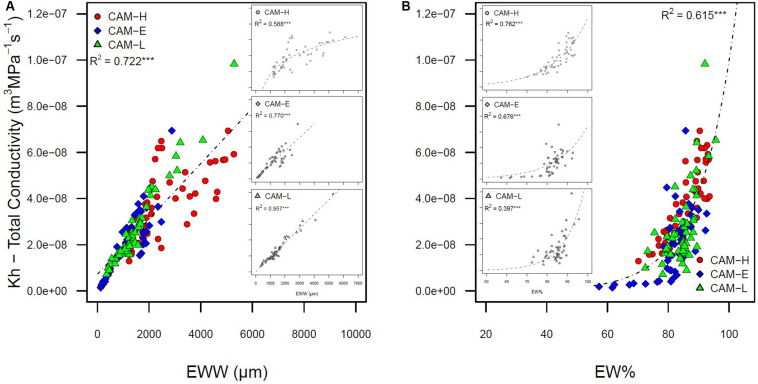
**(A)** Total conductivity (Kh) as a function of earlywood width (EWW). The inset figures express the same relationship for each plot: CAM-H (top), CAM-E (middle), and CAM-L (bottom). A linear function was fitted, except in CAM-H where a logarithmic function shows the highest *R*^2^. **(B)** Total conductivity (Kh) as a function of earlywood percentage (EW%). The inset figures express the same relationship for each plot: CAM-H (top), CAM-E (middle), and CAM-L (bottom). An exponential function was fitted. Each symbol (circle, diamond, and triangle) corresponds to a tree from the three plots CAM-H (red), CAM-E (blue), and CAM-L (green). All relations are significant (***P < 0.001).

Both mean hydraulic diameter (Dh) and specific hydraulic conductivity (Ks) were positively related to tree height (Supplementary Figure S5) while, theoretical hydraulic conductivity (Kh) was not. Although Kh is positively related to Drad (*R*^2^ = 0.503), total number of tracheids (CNO) (*R*^2^ = 0.556), EW% (*R*^2^ = 0.615), basal area increment (BAI) (*R*^2^ = 0.672), TRW (*R*^2^ = 0.707), and EW width (*R*^2^ = 0.722) (all relationships are significant, *P* < 0.001) (Figures 4A,B). In addition high EW% corresponded to larger values of “hydraulic carbon use efficiency” index (HCUE) (*R*^2^ = 0.600, *P* < 0.001) (Supplementary Figure S6). HCUE increased linearly with TRW (*R*^2^ = 0.772, *P* < 0.001) and decreased exponentially with age (*R*^2^ = 0.358, *P* < 0.001), meaning that younger trees had a high carbon investment per unit of water conductivity.

**FIGURE 5:**
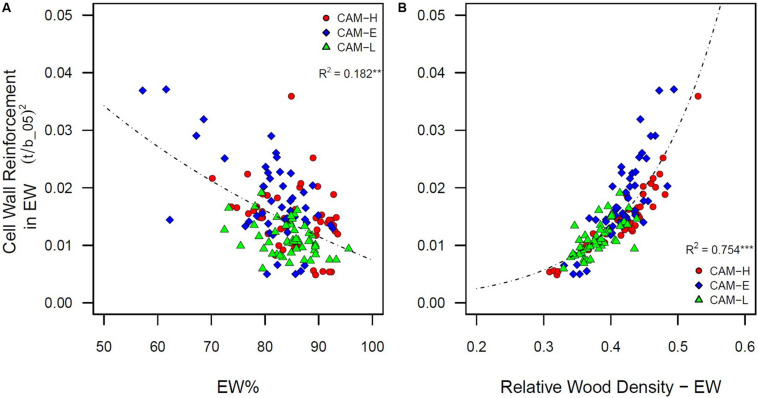
**(A)** Cell wall reinforcement index ([t/b____05_]^2^) in EW portion (5th percentile) as a function of earlywood percentage (EW%). A logarithmic function was fitted. **(B)** Cell wall reinforcement index ([t/b]^2^) in EW portion (5th percentile) as a function of relative wood density in the earlywood. Each symbol (circle, diamond, and triangle) corresponds to a tree from the three plots CAM-H (red), CAM-E (blue), and CAM-L (green). An exponential function was fitted. All relationships are significant (***P < 0.001).

Earlywood percentage (LW%) had a negative (positive) effect on cell wall reinforcement index measured in EW tracheids ([t/b__05_]^2^) (Figure 5A). Trees with denser wood (due to larger LW%, *R*^2^ = 0.638, *P* < 0.001 – Supplementary Figure S7) showed higher values of [t/b]^2^ in the EW portion (Figure 5B). The relative wood density (RWD) was also negatively related to Drad and Dtan (*R*^2^ = 0.618 and *R*^2^ = 0.316, respectively, *P* < 0.001) (Supplementary Figure S7), as well as positively related to CWT (*R*^2^ = 0.526, *P* < 0.001).

**FIGURE 6:**
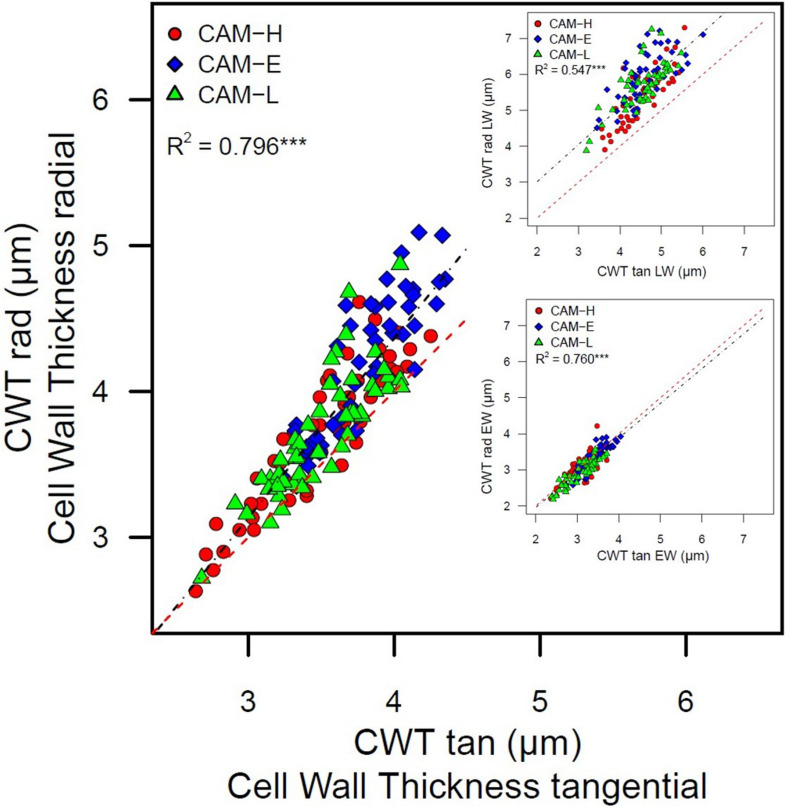
Cell wall thickness in radial direction (CWT rad) as a function of cell wall thickness in tangential (CWT tan) direction. The inset figures represent the same relationship for EW and LW portion. Each symbol (circle, diamond, and triangle) corresponds to trees from the three plots CAM-H (red), CAM-E (blue), and CAM-L (green). The linear regressions are significant (***P < 0.001). The dashed red line represents the 1:1 relationship.

Cell wall thickness in radial cell walls was positively correlated with the CWT in tangential cell walls (Figure 6). In the LW portion of the ring, this relationship showed a higher intercept, as opposed to the 1:1 relationship in the EW, with the increase in tangential direction stronger than in the radial direction. A weak but positive relation between CWT and tree age was also found (*R*^2^ = 0.281, *P* < 0.001), whereas no relation was observed between tree height and CWT.

### Inter-Individual Variation of Wood Anatomical Traits and Their Relationships: Multivariate Analysis

**FIGURE 7:**
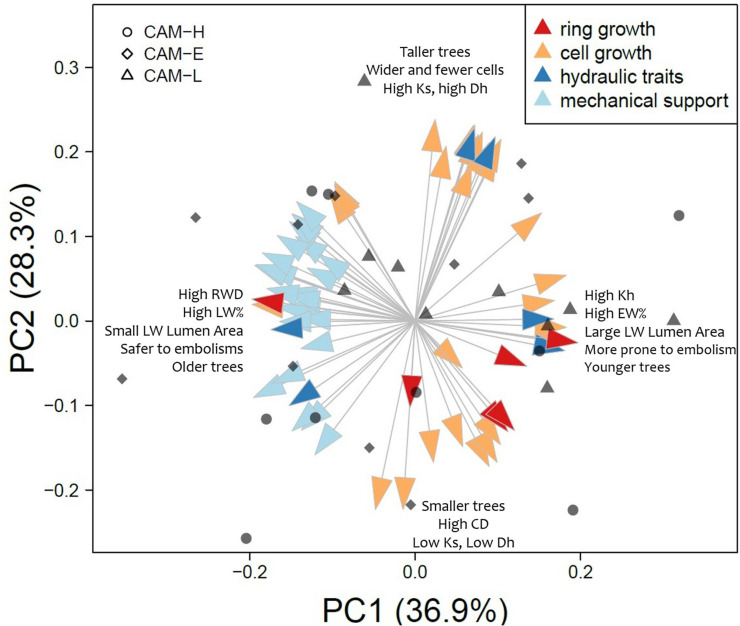
Results of the PCA on all anatomical traits measured for the 2012 tree ring. Arrows represent the eigenvalue of each variable for the first two principal components (PC). Arrow colors indicate the function assigned to each anatomical trait according to the literature (see Table 2). Arrows names are written in Supplementary Figure S8. The three symbols (circle, diamond, and triangle) correspond to individual scores on PC1 (*x*-axis) and PC2 (*y*-axis) from CAM-H, CAM-E, and CAM-L plots, respectively.

A PCA on wood anatomical traits relative to the 2012 tree ring showed that the first three PCs explains the 36.8, 28.5, and 13.3% of variation, respectively (Figure 7). The hydraulic traits Kh and HCUE were correlated positively with PC1, while [t/b]^2^ and [t/b____05_]^2^ negatively (Figure 7, Supplementary Figure S8). The remaining hydraulic traits (Dh and Ks) were positively correlated with PC2, meaning that they were orthogonal with respect to the other hydraulic traits. All mechanical and carbon-allocation traits (Table 2, Supplementary Figure S8) showed a negative correlation with PC1, whereas PC2 was highly correlated with some wood anatomical traits connected to tangential diameter in both EW and LW, and the LA of the EW portion (Figure 7, Supplementary Figure S8).

The results of the multiple regression analysis between the first three PCs and tree height, age and plot were reported in Supplementary Table S1. Scores of PC1 increased with tree height (ß = 0.470, *P* = 0.031) and decreased with age (ß = −0.082, *P* = 0.006; Supplementary Figure S9A). Scores of PC2 increased with tree height (ß = 0.546, *P* < 0.001; Supplementary Figure S9B). Scores of PC3 increased with tree height (ß = 0.218, *P* = 0.037) and were significantly determined by the plot (*P* = 0.006; Supplementary Figure S9C). Scores of PC3 were significantly different between CAM-H and CAM-L (adj *P* = 0.023). Pairwise geographic distances and pairwise differences calculated on PC scores were positively correlated only in CAM-H (*r* = 0.357, *P* = 0.019) (Supplementary Figure S10). This means that, in CAM-H, trees that are spatially closer to each other have similar wood anatomical traits.

### Inter-Annual Variation of Wood Anatomical Traits

**FIGURE 8:**
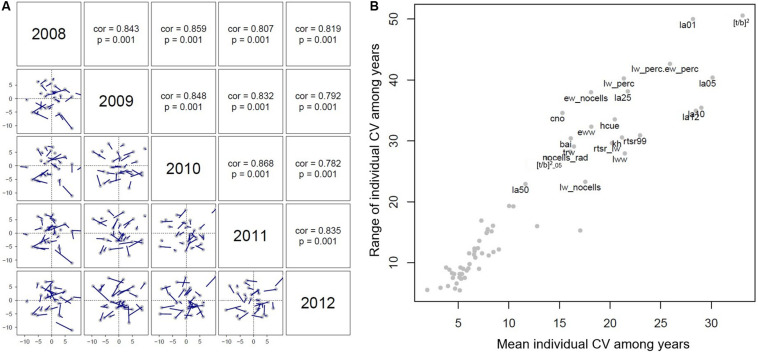
**(A)** Results from the Procrustes test analyzing the stability of PC scores through time. Correlation coefficients and their statistical significance are shown in panels above the diagonal. Blue lines represent shifts in the position of individual trees among years. **(B)** Mean and range of individual coefficient of variation (CV) among years for each anatomical trait. Traits characterized by large variation among years were labeled.

Principal component analysis was performed on the dataset relative to each tree ring (from 2008 to 2012) to test the stability of wood anatomical traits through time. Procrustes tests show consistently that individual PC scores were highly correlated from year to year (*r* ≥ 0.8, *P* = 0.001; Figure 8A). Only 22 out of 65 anatomical traits displayed a mean CV larger than 10% and a range of individual CVs larger than 20% (Figure 8B). The largest inter-annual and inter-individual variation was displayed by [t/b]^2^ and LA of the 1st and 5th percentiles (LW portion) (Table 2, Figure 8B).

## Discussion

### Within a Tree Ring: Ontogenetic Trend and Tracheid Anatomy

Besides confirming that taller trees have larger tracheids in the basal portion of their stem, our results take the discussion forward. EW tracheids in tall trees become larger by increasing both in radial and tangential directions proportionally. However, the tangential diameter of tracheids is partly constrained by the diameter of cambial mother cells and by adjacent tracheids. Despite previous works confirming that tracheid size increases primarily in the radial direction, whereas the tangential diameter and length remain almost constant (Skene, 1972; Cuny et al., 2014), here we suggest that the radial tracheid diameter, and even more their tangential diameter, are primarily influenced by tree height and to a lesser extent by the altitudinal position along the transect. This confirms recent results on conifers growing at the treeline: once the LA of EW tracheids is standardized for tree height, this trait is not related to temperatures (Kašpar et al., 2019).

### Wood Anatomical Variation: Functional Constraints and Trade-Offs

All 65 wood anatomical traits in this study are grouped according to their wood characteristics and function in tree-ring growth, cell growth, hydraulic efficiency and safety, and mechanical support and carbon investment. PCA appears as an efficient analysis for summarizing the information embedded within a large set of wood anatomical traits and shows a high degree of variation both between and within plots (Supplementary Figure S11). We identify two main wood anatomical “strategies” associated with the first and second PCs, respectively (Figure 6). On the one hand, high PC1 scores describe trees with high EW percentages and hydraulic conductivity, whereas low PC1 scores describe trees with high LW% and high RWD, thicker tracheid walls, and high cell wall reinforcement index, traits linked to hydraulic safety. On the other hand, PC2 describes whether each tree invests in tracheid number or in their lumen diameter. High PC2 scores identify trees with few large tracheids, while low PC2 scores identify trees with numerous small ones. Since PCs are orthogonal, this second anatomical strategy is independent to the first one. PC1 is negatively related to tree age showing that older trees tend to have a higher proportion of LW, thicker cell wall in both radial and tangential direction, and higher LW RWD than younger trees, which may limit their water transport capacity, increase the investment in carbon and increase their resistance to embolism (Domec and Gartner, 2002; Rosner et al., 2014) as explained in the following section. PC2 is strongly, positively related to tree height, meaning that taller trees tend to have fewer but larger tracheids to maximize water conductivity per unit area. Tall trees have a high hydraulic tracheid diameter and Ks at breast height (Rosner et al., 2008; Anfodillo et al., 2012; Olson et al., 2014), whereas the Kh is mainly related to the amount of EW: the higher the EW percentage, the higher is the conductivity.

Recent studies have shown a plant size-dependent sensitivity to drought (Bennett et al., 2015; Fajardo et al., 2018; Stovall et al., 2019). Bennett et al. (2015) found that drought has more detrimental impacts on the stem diameter growth of large trees. Stovall et al. (2019) reported that tree height is the most important predictor of tree death during drought episodes, with tall trees (>30 m) dying at a rate of more than double than short trees (<15 m). However, tall or large trees are not always old trees, and the lack of such correlation depends on species characteristics (e.g., shade-tolerant vs light-tolerant species) and within-population structure, such as local site conditions, tree’s disturbance history, growth form, as well as individual genotype (Lindenmayer and Laurance, 2016). Finally, the site conditions can play an important role in wood anatomical trait variability. A significant plot-effect is found on PC3; in particular, a statistically significant difference between PC3 scores of CAM-H and CAM-L. This difference may suggest an altitudinal effect as these two plots are the most contrasting in terms of elevation (1,730 vs 1,475 m). However, PC3 explained just 13.3% of the total inter-individual wood anatomical variation, suggesting that the environment is, in any case, less relevant than tree height and age in affecting wood anatomical trait variation. This could be partially related to the limited extension of the altitudinal gradient explored (∼300 m) that, however, encompasses the elevation gradient of spruce in the study area. For this reason, it could be worth exploring the inter-individual variation of wood anatomical traits along larger and replicated altitudinal transects and in more limiting environmental conditions. A weak but statistically significant spatial effect is found in CAM-H, where neighbor trees display more similar anatomical traits. In this case, spatial proximity is considered as a proxy for micro-environmental heterogeneity. Avanzi et al. (2019) demonstrated the relevance of micro-environmental heterogeneity of growth patterns on a larger dendrochronological dataset of trees sampled in the same plots. In this study, we analyzed 10 trees per plot, not a high number for fully capturing the micro-environmental heterogeneity.

### Wood Anatomical Traits at the Inter-Individual and Inter-Annual Level

Our analysis on inter-annual variation of wood anatomical traits investigates which traits are likely to show no or small changes through time. PCA results are highly correlated among years, meaning that overall trees have similar wood anatomical traits in their last formed five rings. This result emphasizes, over a limited temporal window, how trees exhibit globally coherent wood anatomical patterns, even with different mean temperatures and total precipitation of the growing season (Supplementary Figure S12). Nonetheless, the coefficients of variation for each single wood anatomical trait reveal that the magnitude of such inter-annual variation changes among traits and individuals. We found that the cell wall reinforcement index of the entire ring, the LA of LW, and LW% are the most variable traits both at inter- and intra-individual level. LW is probably the wood anatomical trait mostly involved in potentially adaptive responses to climate and environmental changes. According to Larson (1969), EW to LW ratio is influenced by tree crown size that, in turn, depends on stand density and competition. When growing conditions are ideal, height growth is enhanced and the LW% is lowered (Domec and Gartner, 2002). In our study site, the EW percentage is on average >80%, pointing toward a weak effect of climate on individual tree growth dynamics, as shown by a climate-growth correlation analysis performed on a larger dataset of trees sampled from the same plots (Avanzi et al., 2019).

### Wood Density and Hydraulic Vulnerability

Latewood percentage affects RWD and also the hydraulic properties of coniferous wood (Hacke et al., 2001; Domec and Gartner, 2002). In Norway spruce, EW tracheids are the most effective water-conducting pipes with higher numbers of pits per conduit length unit compared to LW tracheids (Rosner, 2013). Wood density shows moderate to high values of heritability in conifers (e.g., Hannrup et al., 2004; Martinez Meier et al., 2008; Chen et al., 2018) and, despite that this trait is not directly linked to hydraulic vulnerability, it is involved in mechanisms of drought tolerance and hydraulic failure, with higher wood density being associated with lower vulnerability to cavitation (e.g., Dalla-Salda et al., 2009; Rosner, 2013; Rosner et al., 2016; Fernández et al., 2019). As in previous studies (Bouche et al., 2014; Bouriaud et al., 2015), the present results show that RWD in LW is more related to tree age than to tree height, and that trees with higher RWD in LW feature a higher cell wall reinforcement index. We used the cell wall reinforcement index as a proxy for hydraulic vulnerability (e.g., Hacke et al., 2001; Rosner, 2013), concluding that trees with high RWD are more resistant to tracheid embolism. Studies on LW, EW, and transition-wood tracheids suggest that, when a water-deficit episode takes place, cavitation first occurs and expands in the LW tracheids. Only when water deficit further increases, embolism spreads in the EW and finally reaches the transition wood (Dalla-Salda et al., 2014). However, the loss in conductivity is not related to where the initial cavitation occurs, but to the wood anatomical structure (e.g., RWD and EW to LW ratio) that shapes the species vulnerability to drought events (Luss et al., 2019).

### Implications for Ecological and Biological Studies

In conclusion, our findings seem to consistently demonstrate that it is crucial in any wood anatomical, ecological, and evolutionary genetics study to record and consider tree height to correctly investigate inter-individual wood anatomical variation. By doing so, wood anatomical traits can be standardized based on tree height as opposed to tree diameter which is not always directly related to tree age. Additionally, we also consider crucial to account for age-related trends that might affect wood anatomical traits with mechanical support functions, such as CWT, RWD, and lumen diameter of LW tracheids. Such traits can ultimately affect tree resistance to embolism. We recommend that analyzing wood anatomical traits within few tree rings but from a large number of individuals is a good method for capturing most of the variance in both wood anatomical traits and microenvironmental conditions. This strategy may avoid the “noise” created by difference in tree height, and age. Finally, the creation of a common garden would be extremely useful to determine whether and how individual genotypes drive wood anatomical variation, minimizing microsite heterogeneity.

## Data Availability Statement

The datasets generated for this study are available on request to the corresponding author.

## Author Contributions

All authors planned and designed the research. AlP, AnP, CA, and CU conducted the fieldwork. AlP collected the data. AlP, GA, AnP, CA, and AC analyzed the data. AlP led manuscript writing with contributions from all authors. All authors contributed critically to the drafts and gave final approval for publication.

## Conflict of Interest

The authors declare that the research was conducted in the absence of any commercial or financial relationships that could be construed as a potential conflict of interest.

## Footnotes

1 https://climexp.knmi.nl
