# Supplementary material for: Functional Relationships of Wood Anatomical Traits in Norway Spruce
Source: Front Plant Sci. 2020 May 26;11:683. doi: 10.3389/fpls.2020.00683 (PMC7266088; doi:10.3389/fpls.2020.00683)
Supplement: Supplementary file 1 [file Data_Sheet_1.docx]

**Appendices**

**
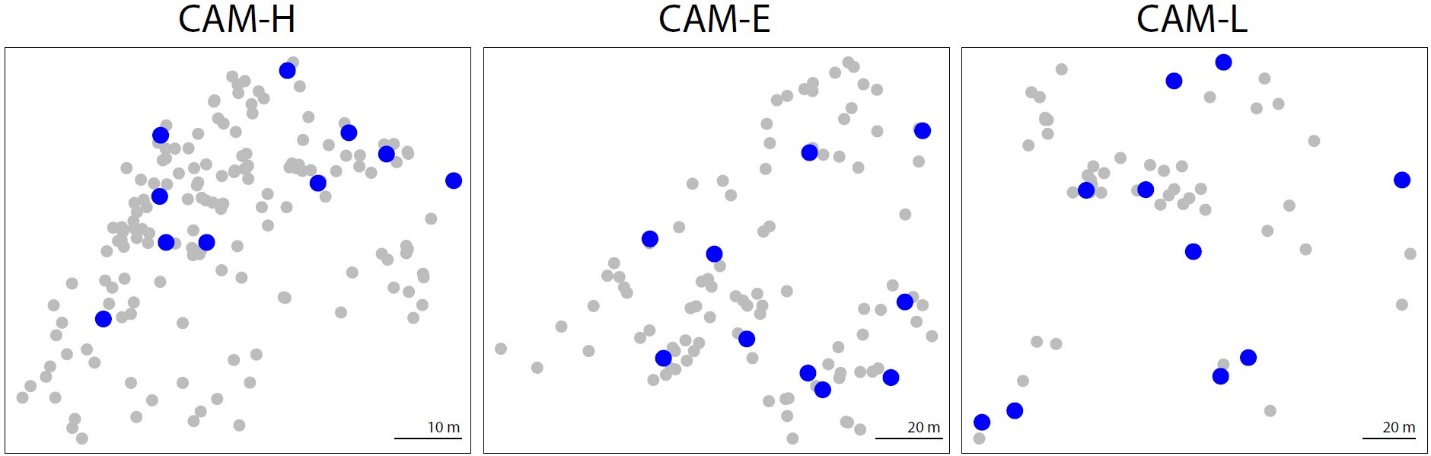
**

**Fig. S1.** Spatial proximity of trees selected for this study (in blue). Trees selection maximize the variability on tree height and tree age. Grey dots correspond to trees sampled in the studied area previously investigated by Avanzi *et al.* (2019). The bar scale differs from the three plots.

**
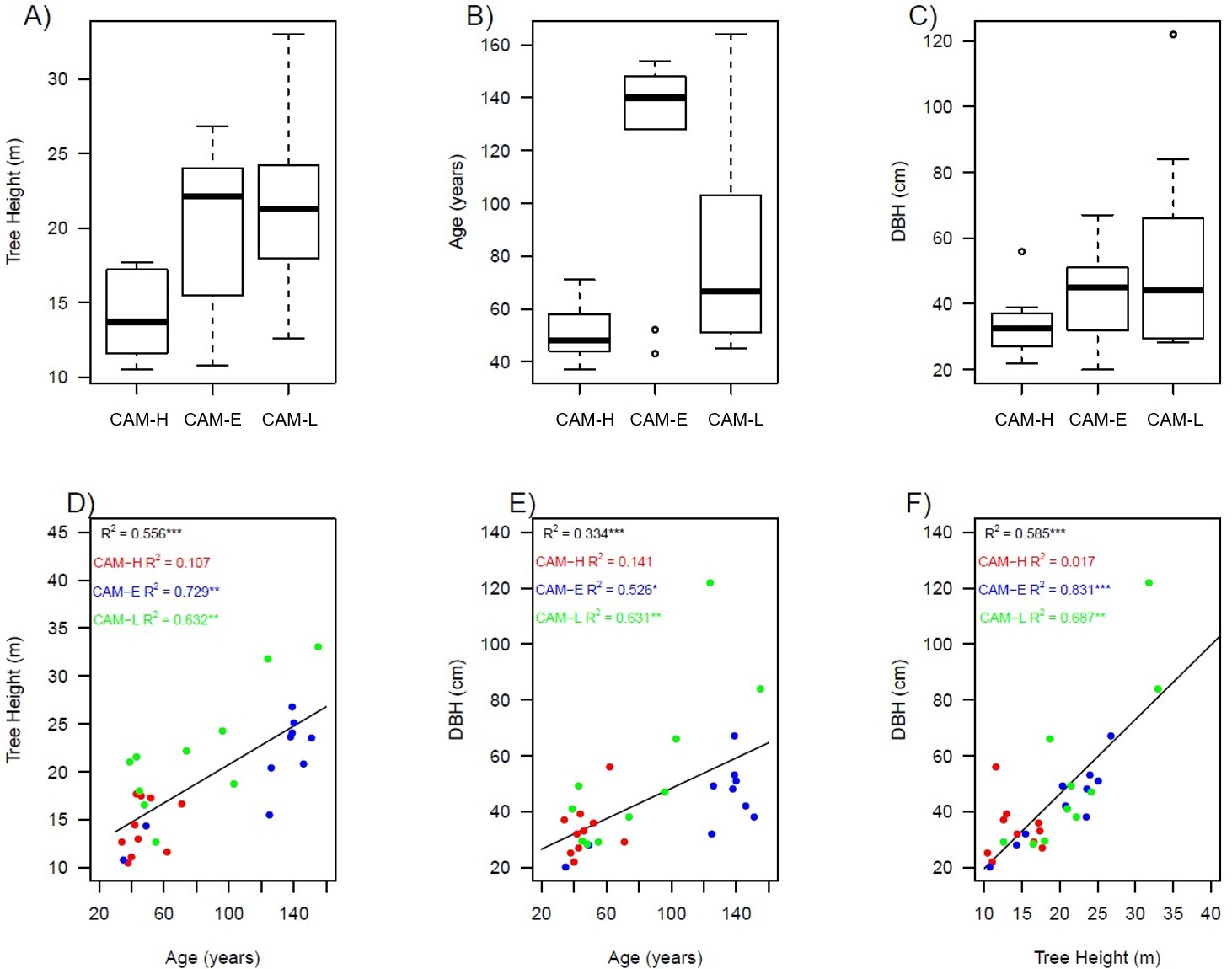
**

**Fig. S2.** Box plot showing the variability in **A)** Tree height, **B)** Age and **C)** DBH for each plot: CAM-H (high), CAM-E (intermediate) and CAM-L (low) plot, and their relationship (**D-F**). Tree heights ranged from 10 to 33 meters, tree ages spanned from 34 to 155 years, and DHB from 22 to 122 cm. The horizontal line in the box plot represents the median. Younger trees were mainly found at the high-altitude plot (CAM-H), close to the forest limit, while older trees were mainly found at the intermediate plot (CAM-E). Overall tree height and age were positively correlated (r = 0.745, P < 0.001). However, at the plot level, high significant correlations between age and height were found in CAM-E (r = 0.854, P < 0.01) and CAM-L (r = 0.795, P < 0.01), but not in CAM-H (r = 0.327, P = 0.356). Similarly, tree height and DBH were positively correlated only in CAM-E (r = 0.911, P < 0.01) and CAM-L (r = 0.828, P < 0.01), but not in CAM-H (r = -0.13, P = 0.72).

**
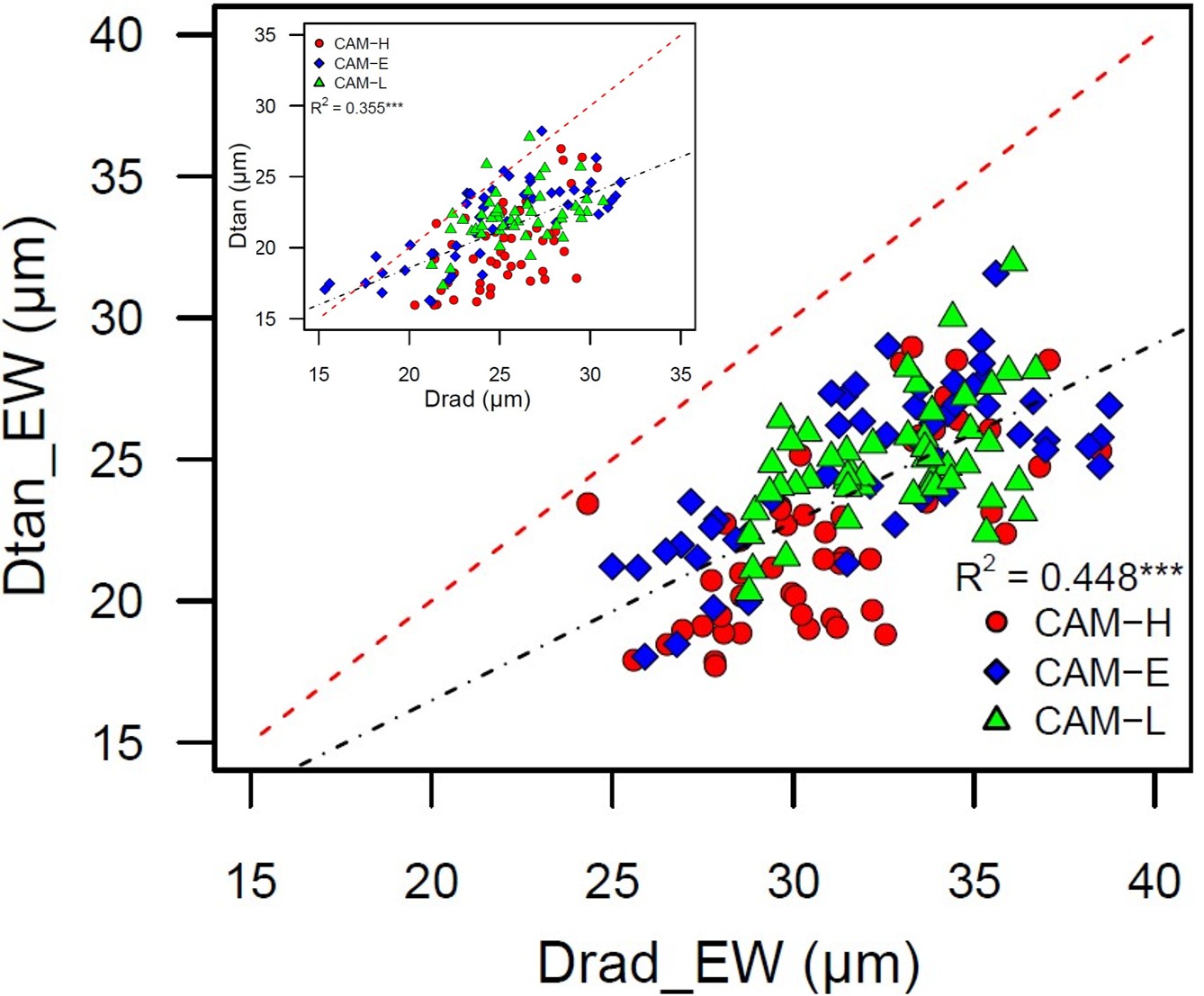
**

**Fig. S3.** Tangential diameter (Dtan EW) as a function of radial diameter (Dtan EW) of earlywood tracheids. The inset figure represents the same relation considering the Drad and Dtan of the entire ring (earlywood and latewood). Each symbol (circle, diamond and triangle) corresponds to a tree from the three plots CAM-H (red), CAM-E (blue) and CAM-L (green). The linear regression is positive and significant (^***^P < 0.001). The dashed red line represents the 1:1 relationship.


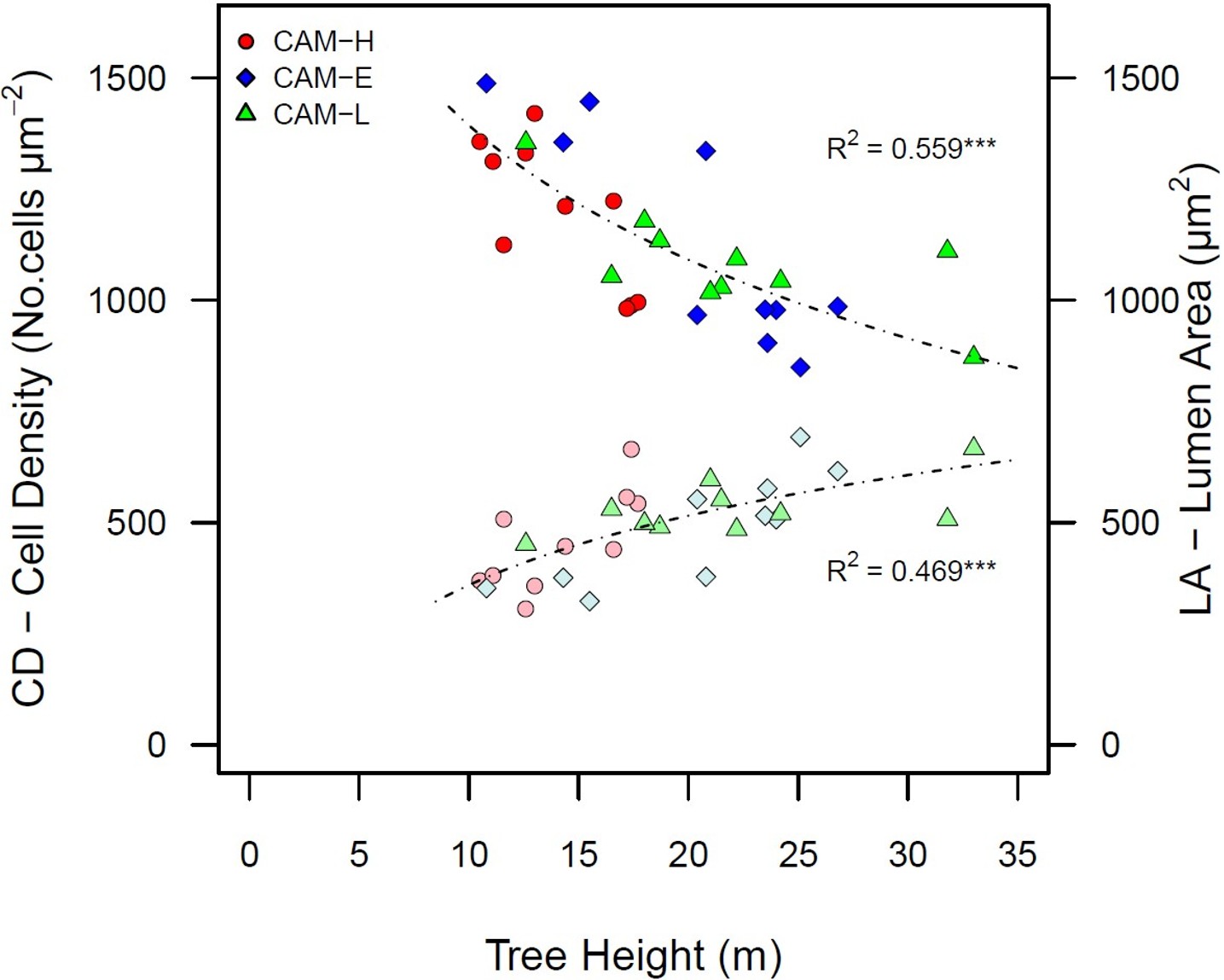


**Fig. S4.** Cell density (CD) highlighted in bright colour as a function of tree height, and lumen area (LA) highlighted in pale colour as a function of tree height. The data shown are only for the outmost year 2012. Each symbol (circle, diamond and triangle) corresponds to a tree from the three plots CAM-H (red), CAM-E (blue) and CAM-L (green). A logarithmic function was fitted in both CD and LA parameters and shows opposite trend: negative between CD and tree height, and positive between LA and height. The logarithmic regressions are significant (^***^P < 0.001).


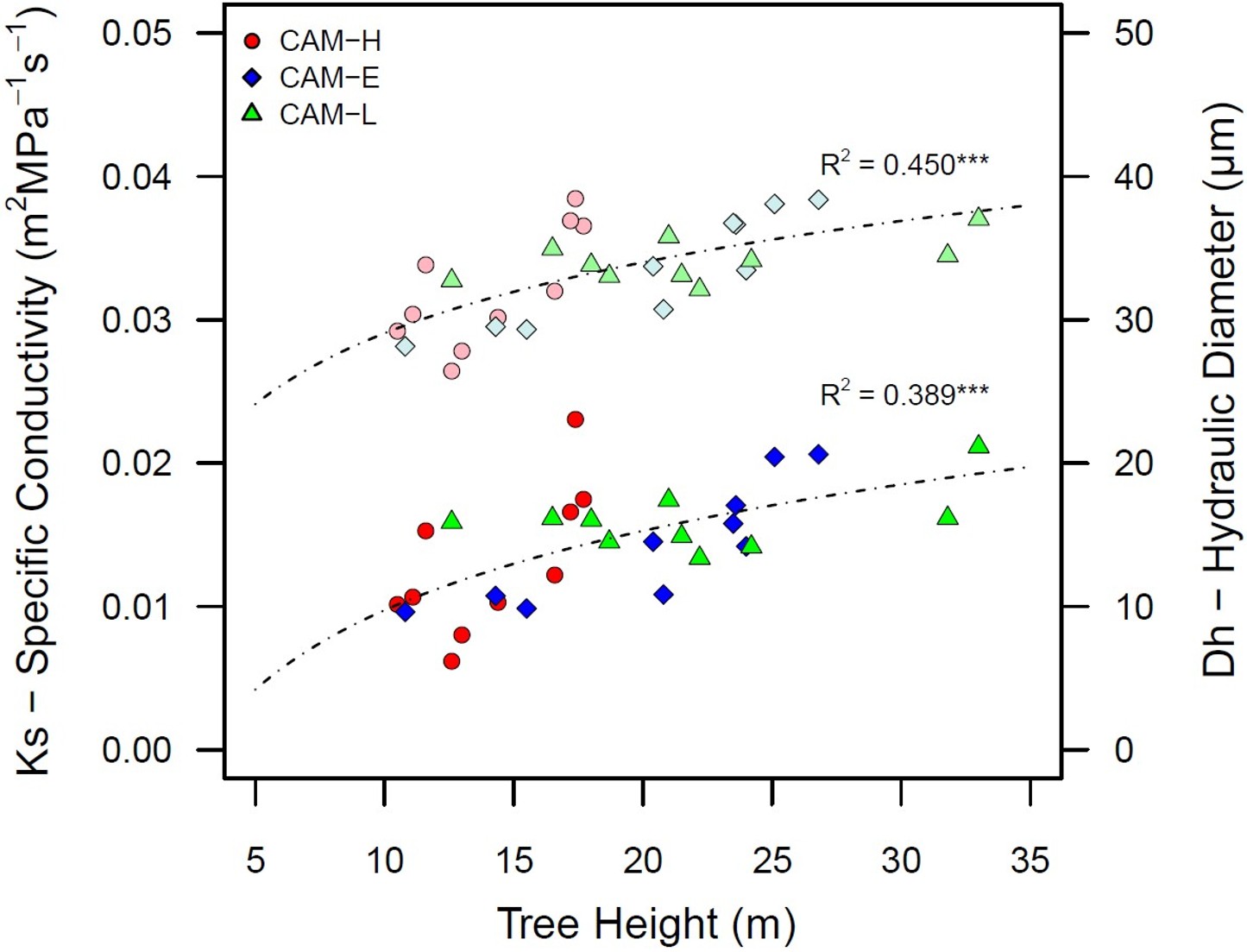


**Fig. S5.** Specific conductivity (Ks) highlighted in bright colour as a function of tree height, and mean hydraulic diameter (Dh) highlighted in pale colour as a function of tree height. The data shown are only for the outmost year 2012. Each symbol (circle, diamond and triangle) corresponds to a tree from the three plots CAM-H (red), CAM-E (blue) and CAM-L (green). A logarithmic function was fitted in both CD and LA parameters, showing a positive relationship. The logarithmic regressions are significant (P < 0.001).

**
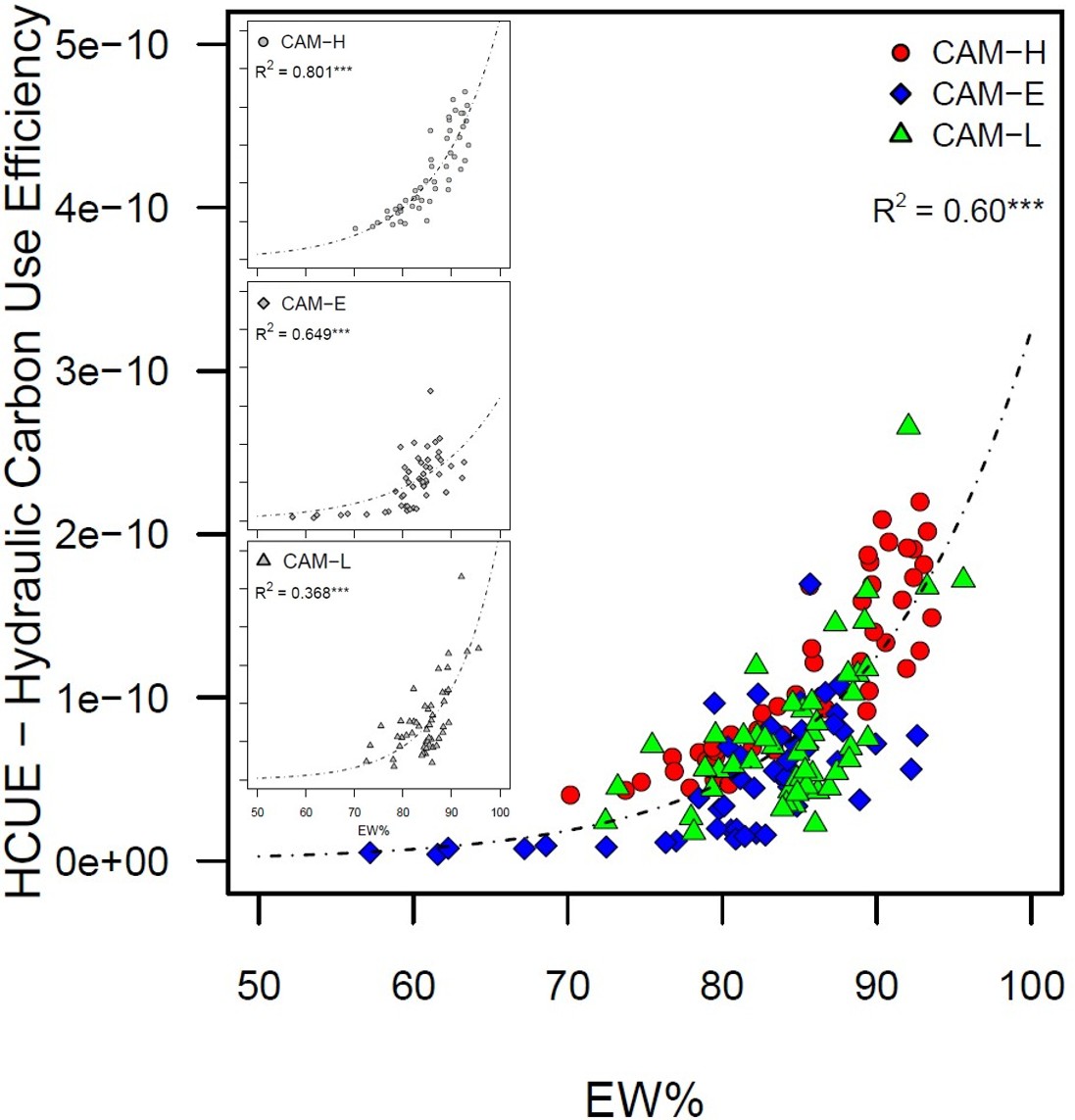
**

**Fig. S6.** HCUE index as a function of earlywood percentage (EW%). The inset figures express the same relationship for each plot: CAM-H (top), CAM-E (middle) and CAM-L (bottom). Each symbol (circle, diamond and triangle) corresponds to a tree from the three plots CAM-H (red), CAM-E (blue) and CAM-L (green). An exponential function was fitted. The exponential functions are significant (^***^P < 0.001).

**
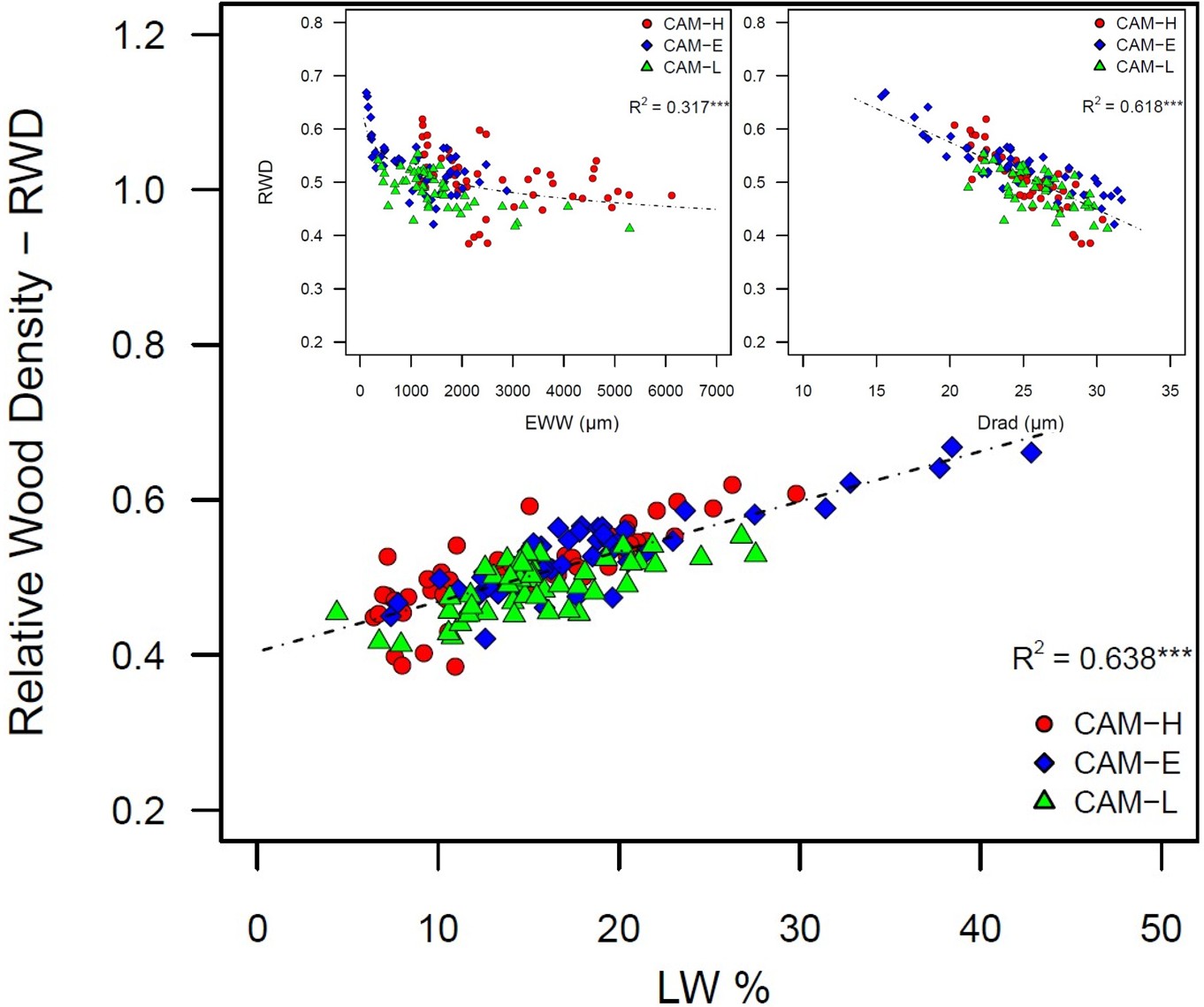
**

**Fig. S7.** Relative wood density (RWD) of the entire ring as a function of latewood percentage (LW%). The inset figures express the relation between RWD and earlywood width (EWW), and between RWD and radial diameter (Drad). Each symbol (circle, diamond and triangle) corresponds to a tree from the three plots CAM-H (red), CAM-E (blue) and CAM-L (green). Linear functions were fitted, except between RWD and EWW where a logarithmic function was fitted. All relations are significant (^***^P < 0.001).


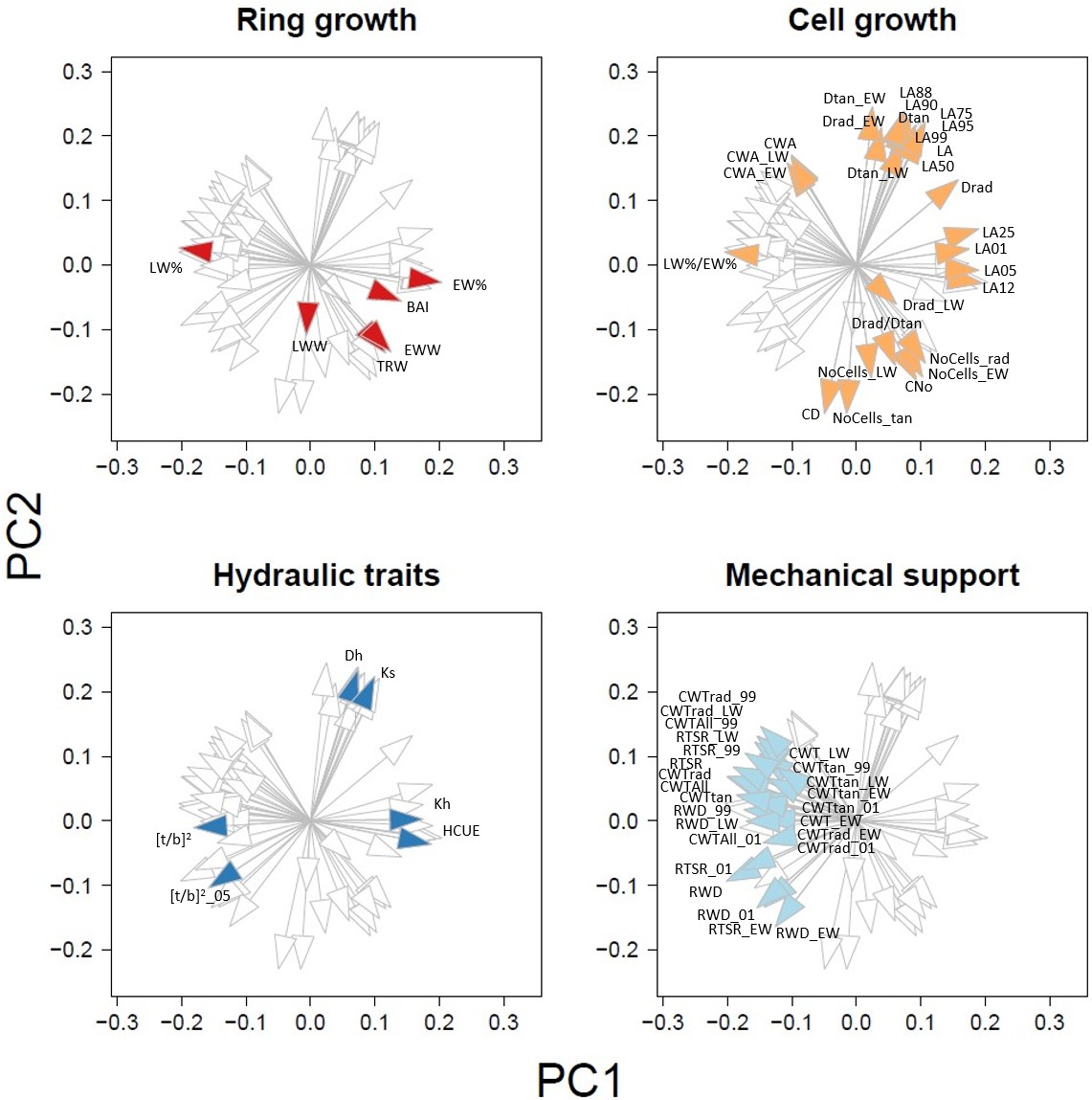


**Fig. S8.** Results of PCA on all anatomical traits measured for the 2012 tree ring, divided in the four functional traits: ring growth, cell growth, hydraulic traits (safety and efficiency), and mechanical support. Arrows represent the eigenvalue of each variable for the first two PCs. Arrows label of each trait is expressed. For some anatomical traits, different percentile is calculated (See **Table 2**).


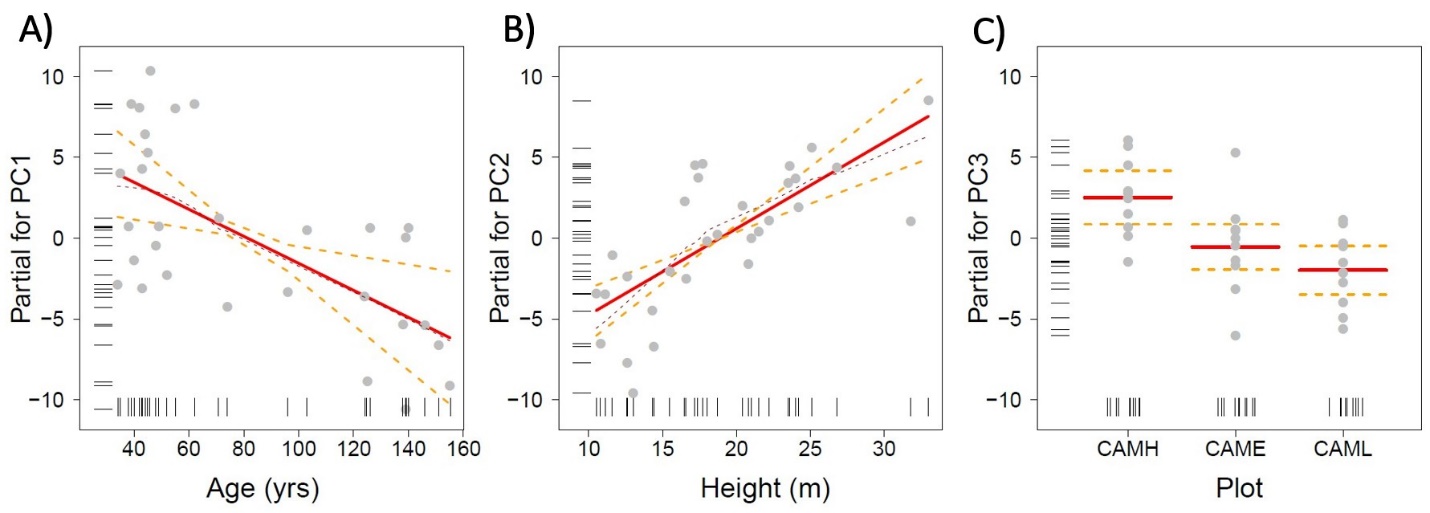


**Fig. S9.** Results of the multiple regression of each of the first three PCs against tree height, age and plot. For each PC, only the statistically most significant effect was graphically represented, which was **A)** tree age, **B)** tree height, and **C)** plot, respectively.


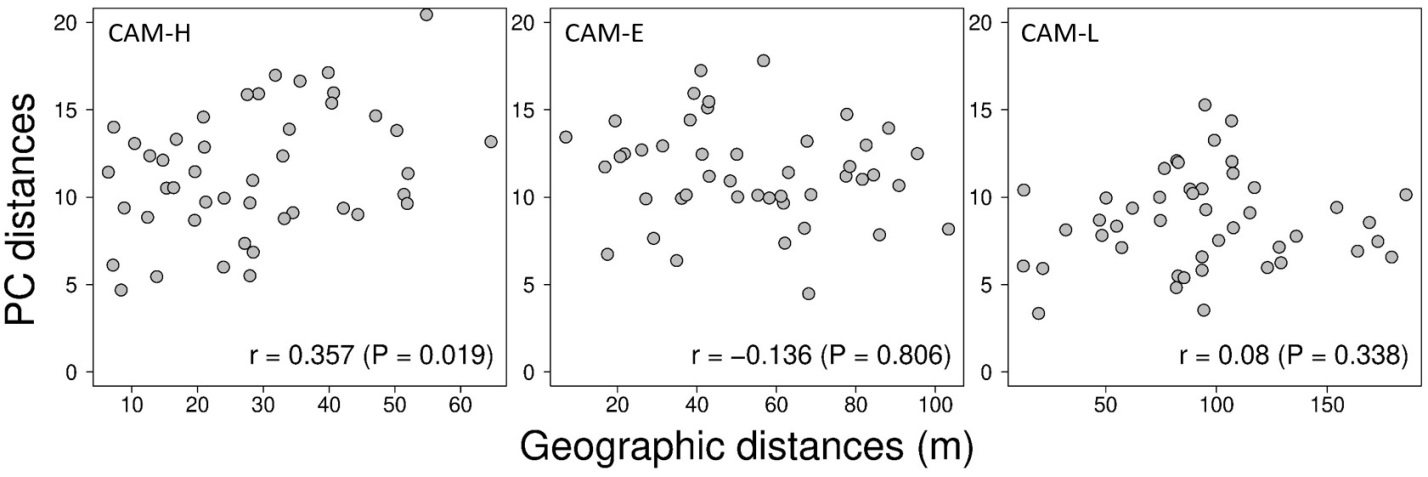


**Fig. S10.** Scatterplot of pairwise geographic distances (on the x-axis) vs pairwise distances calculated from individual PC scores (on the y-axis). Mantel test was positive and significant only in CAM-H (high-altitude plot).


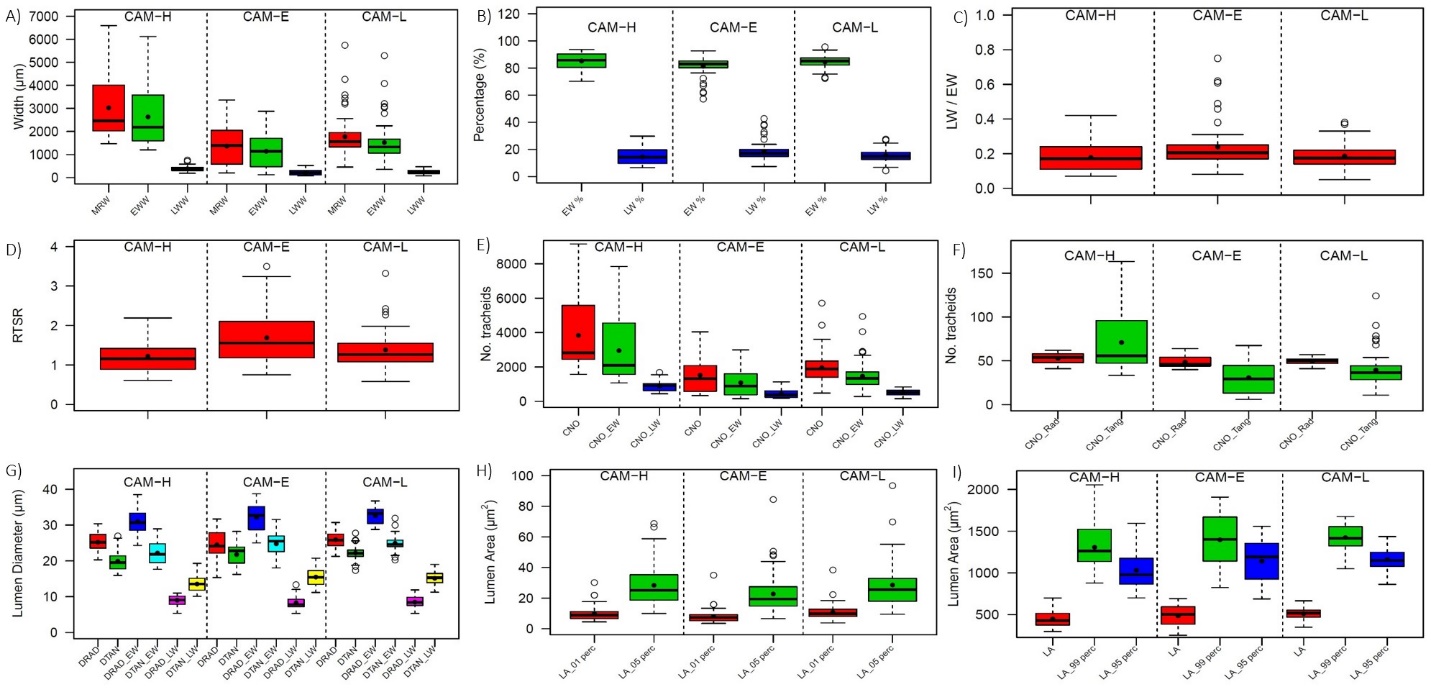

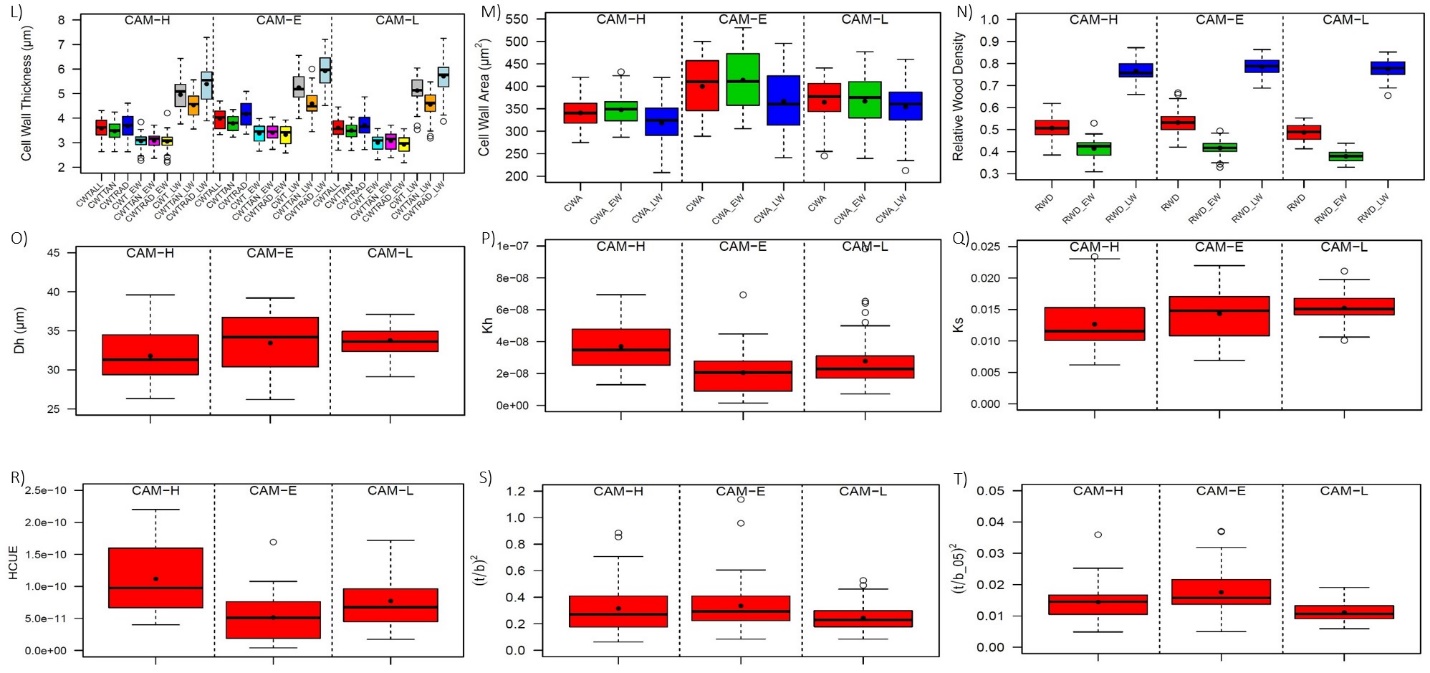


**Fig. S11.** Box plot of each anatomical trait per plot: CAM-H, CAM-E and CAM-L. **A)** Tree-ring width (TRW), EW width and LW width; **B)** LW and EW percentage; **C)** LW and EW ratio; **D)** RTSR (Mork’s index); **E)** Number of tracheids of the entire ring and divided in EW and LW; **F)** Number of tracheids in radial and tangential direction; **G)** Lumen diameter divided in EW and LW, and in radial and tangential direction; **H-I)** Lumen area at different percentile representing the EW and LW portion; **L)** Cell wall thickness divided in EW and LW, and in radial and tangential direction; **M)** Cell wall area of the entire ring and divided in EW and LW; **N)** Relative wood density of the entire ring and divided in EW and LW; **O)** Hydraulic Diameter (Dh); P) Total hydraulic conductivity; **Q)** Specific conductivity; **R)** Hydraulic Carbon Use Efficiency (HCUE); **S)** Cell wall reinforcement [(t/b)^2^] of the entire ring; and **T)** Cell wall reinforcement in the earlywood portion [(t/b_05)^2^]. In each box plot, the black line represents the median and the dot the mean.


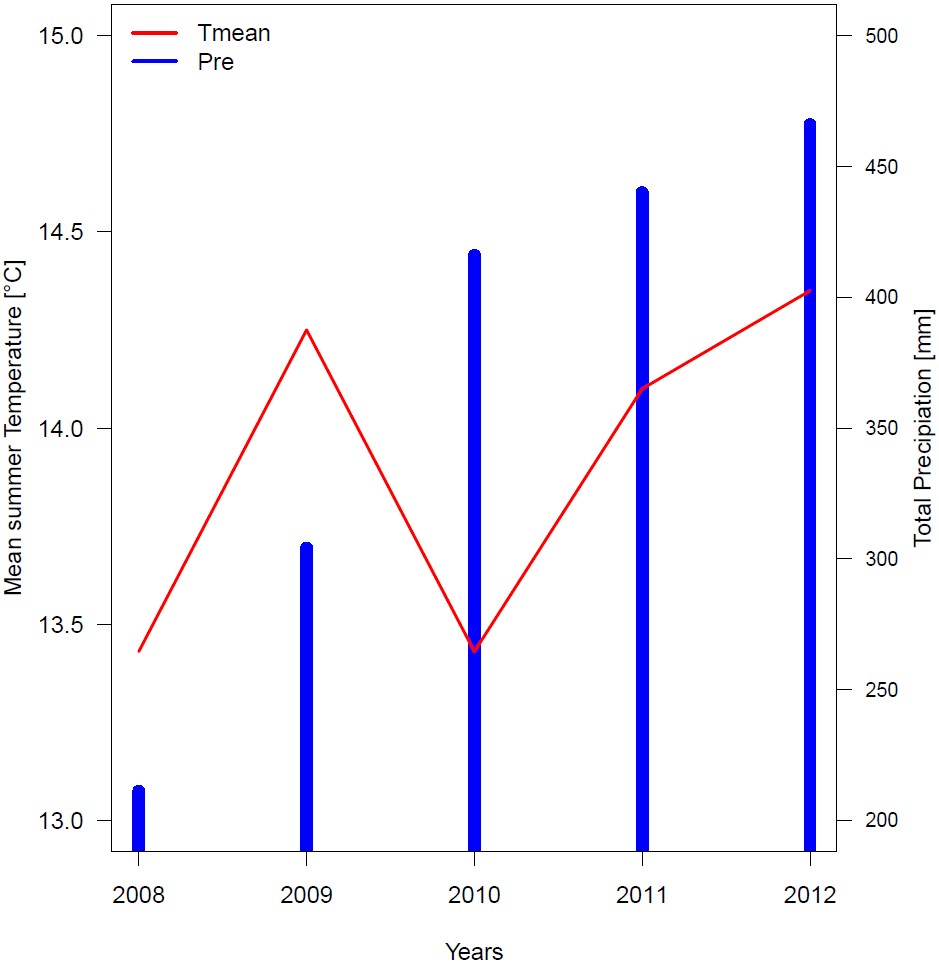


**Fig. S12.** Mean monthly temperatures (red line) and total monthly precipitation (blue bar) during the growing season (from June to September) of the five years (2008-2012) analysed.

**Table S1.** ANOVA results of the multiple regression analysis of the first three PCs with the variables tree height, age and plot.

|  | **PC1** |  |  |  | **PC2** |  |  |  | **PC3** |  |  |  |
| --- | --- | --- | --- | --- | --- | --- | --- | --- | --- | --- | --- | --- |
|  | ***Sum sq*** | ***Df*** | ***F value*** | ***P*** | ***Sum sq*** | ***Df*** | ***F value*** | ***P*** | ***Sum sq*** | *Df* | *F value* | *P* |
| **Height** | 106.31 | 1 | 5.50 | 0.027 | 293.04 | 1 | 33.65 | < 0.001 | 42.41 | 1 | 6.70 | 0.016 |
| **Age** | 171.98 | 1 | 8.90 | 0.006 | - | - | - | - | 18.79 | 1 | 2.97 | 0.097 |
| **Plot** | - | - | - | - | - | - | - | - | 84.25 | 2 | 6.66 | 0.005 |
| **Residuals** | 521.94 | 27 |  |  | 243.84 | 28 |  |  | 158.16 | 25 |  |  |
